# Pneumocystis jirovecii pneumonia associated with immune checkpoint inhibitors: A systematic literature review of published case reports and disproportionality analysis based on the FAERS database

**DOI:** 10.3389/fphar.2023.1129730

**Published:** 2023-03-15

**Authors:** Shuang Xia, Hui Gong, Yi-kun Wang, Ling Liu, Yi-chang Zhao, Lin Guo, Bi-kui Zhang, Mayur Sarangdhar, Yoshihiro Noguchi, Miao Yan

**Affiliations:** ^1^ Department of Pharmacy, The Second Xiangya Hospital, Central South University, Hunan, China; ^2^ International Research Center for Precision Medicine, Transformative Technology and Software Services, Hunan, China; ^3^ Toxicology Counseling Center of Hunan Province, Hunan, China; ^4^ Hunan University of Chinese Medicine, Hunan, China; ^5^ Division of Biomedical Informatics, Cincinnati Children’s Hospital Medical Center, Cincinnati, OH, United States; ^6^ Division of Oncology, Cincinnati Children’s Hospital Medical Center, Cincinnati, OH, United States; ^7^ Department of Pediatrics, University of Cincinnati College of Medicine, Cincinnati, OH, United States; ^8^ Laboratory of Clinical Pharmacy, Gifu Pharmaceutical University, Gifu, Japan

**Keywords:** immune checkpoint inhibitors, pneumocystis jirovecii pneumonia, pharmacovigilance, FAERS, systematic literature review

## Abstract

**Background:** Pneumocystis jirovecii pneumonia (PJP) has been reported with ICIs but limited to case reports. The clinical features of PJP with ICIs remain mostly unknown. This study aims to investigate the association of PJP with ICIs and describe clinical features.

**Methods:** Reports of PJP recorded in FAERS (January 2004–December 2022) were identified through the preferred term “Pneumocystis jirovecii pneumonia”. Demographic and clinical features were described, and disproportionality signals were assessed through the Reporting Odds Ratio (ROR) and Information Component (IC), using traditional chemotherapy and targeted therapy as comparators, and adjusting signals by excluding contaminant immunosuppressive drugs and pre-existing diseases. A systematic literature review was conducted to describe clinical features of published PJP reports with ICIs. Bradford Hill criteria was adopted for global assessment of the evidence.

**Results:** We identified 677 reports of PJP associated with ICIs, in which 300 (44.3%) PJP cases with fatal outcome. Nivolumab (IC_025_ 2.05), pembrolizumab (IC_025_ 1.88), ipilimumab (IC_025_ 1.43), atezolizumab (IC_025_ 0.36), durvalumab (IC_025_ 1.65), nivolumab plus ipilimumab (IC_025_ 1.59) have significant signals compared to other drugs in FAERS database. After excluding pre-existing diseases and immunosuppressive agents which may increase susceptibility of PJP, the signals for PJP associated with nivolumab, pembrolizumab, durvalumab, nivolumab plus ipilimumab remained robust (IC_025_ > 0). When compared to other anticancer regimens, although all ICIs showed a lower disproportionate signal for PJP than chemotherapy, nivolumab (IC025 0.33, *p* < 0.001), pembrolizumab (IC025 0.16, *p* < 0.001), both PD-1 inhibitors, presented a higher signal for PJP than targeted therapy. Male gender (IC_025_ 0.26, *p* < 0.001) and age >65 years (IC_025_ 0.38, *p* < 0.001) were predominant in PJP cases associated with across all ICIs. In literature, 15 PJP cases associated with ICIs were reported in 10 published case reports. 12 of 15 (80.0%) of cases received PD-1 inhibitors before PJP was diagnosed.

**Conclusion:** By the combined analysis of post-marketing data from FAERS and published case reports, we identified ICIs may be associated with PJP, especially in males aged >65years. After accounting for confounders, PD-1 inhibitors emerged with a robust disproportionality signal when compared to PD-L1/CTLA-4 inhibitors as well as targeted therapy. Further research is warranted to validate our findings.

## 1 Introduction

Immune checkpoint inhibitors (ICIs) have dramatically changed the treatment landscape of many types of malignancies. As of November 2022, FDA has approved 10 ICI agents including PD-1 inhibitors: nivolumab, pembrolizumab, cemiplimab, dostarlimab, PD-L1 inhibitors: atezolizumab, avelumab, durvalumab, CTLA-4 inhibitors: ipilimumab and tremelimumab, LAG-3 inhibitors: relatlimab. These ICIs generate durable efficacy in certain types of cancer, however, there is a significant concern ICIs-mediated toxicities. Toxic effects from these ICI agents are related to removing nodes of self-tolerance and unleashing autoimmune-like phenomena (Postow et al., 2018). Although usually manageable with corticosteroid and immunosuppressants administration, clinically severe events leading to morbidity and even mortality may complicate ICI treatment (Wang et al., 2018).

Pneumocystis jirovecii pneumonia is a form of pneumonia that is caused by the yeast-like fungus Pneumocystis jirovecii. A recent study (Lee et al., 2019) showed that the 3-month mortality rate of lung cancer patients with the PJP was as high as 61.6%, suggesting the severity of PJP infection in lung cancer patients. Moreover, the Infectious Diseases Working Party (AGIHO) of the German Society for Hematology and Medical Oncology (DGHO) recommended that primary prophylaxis of Pneumocystis jirovecii pneumonia in patients with hematologic malignancies and solid tumors are warranted (Classen et al., 2021). To the best of our knowledge, only a few case reports/series have described Pneumocystis jirovecii pneumonia (PJP) during pembrolizumab (Liu et al., 2020; Sadek et al., 2020; Rath et al., 2021; Si et al., 2021; Hiba et al., 2022; Sanka and Hsu, 2023), nivolumab (Schwarz et al., 2019), ipilimumab (Arriola et al., 2015; Finbar Slevin et al., 2016; Sadek et al., 2020), nivolumab plus ipilimumab (Moujaess et al., 2020) and other anti-PD-1 inhibitors (Liu et al., 2020; Feng et al., 2021).

However, case reports can only provide a partial epidemiological perspective. There are no meta-analysis or systematic reviews to investigate the association of PJP and ICIs. In addition, the clinical manifestations of PJP in patients on ICIs are not well known. The analysis of spontaneous reporting databases, such as the FDA Adverse Even Reporting System (FAERS) and WHO Vigibase, allows a broader perspective by collecting unpublished reports of adverse events submitted all over the world occurring in real-world unselected subjects with comorbidities, poly-pharmacotherapy and complex anticancer combination regimens (Raschi et al., 2020), which ensures rapid detection of even rare adverse events. Previous disproportionality analysis based on the FAERS database indicated that interstitial lung disease (Guo et al., 2023), pulmonary tuberculosis (Zhu et al., 2022a) and pneumonitis (Cui et al., 2022) were found to be significantly associated with ICIs exposure. But it is still unclear if ICIs were associated with increased reporting frequency of PJP in the real-world clinical setting.

Herein, we conducted a large-scale pharmacovigilance study by using the US Food and Drug Administration (FDA) Adverse Event Reporting System (FAERS) database to investigate the link between PJP and ICIs. A systematic literature review was conducted to incorporate the information from published papers and compare with post-marketing data from FAERS database.

## 2 Materials and methods

### 2.1 Data sources and study design

**FIGURE 1:**
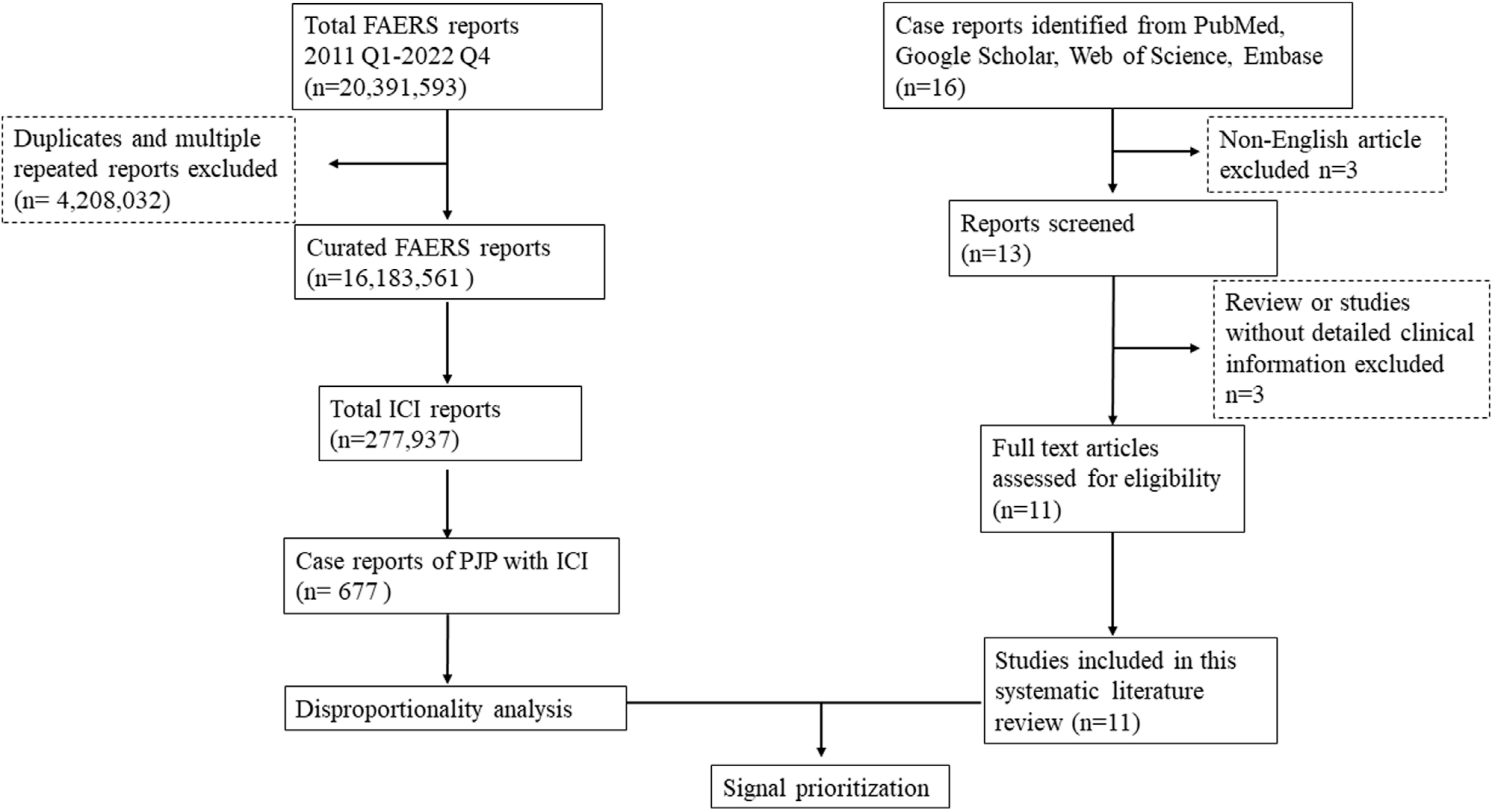
Flow chart to combine the FAERS disproportionality analysis with the systematic literature review. FAERS, FDA Adverse Event Reporting System; ICI, immune checkpoint inhibitor; Q1, quarter 1; PJP, Pneumocystis jirovecii pneumonia.

FAERS is one of the largest publicly available databases designed to support the United States Food and Drug Administration (FDA) post-marketing safety surveillance program, which gathering more than 20 million reports worldwide, including the United States, Europe, and Asia. It allows for the signal detection and quantification of the association between drugs and reporting of AEs. AERS*Mine* is a validated multi-cohort analyzing application designed to mine data across millions of patient reports (currently 19,089,556) from the FDA’s Adverse Event Reporting System (Sarangdhar et al., 2016).A recent high-impact study which combined clinical cardiotoxicity of kinase inhibitors with cell line-derived transcriptomic datasets to identify a gene signature that can predict risk of cardiotoxicity by leveraging AERS*Mine* to visit FAERS data (van Hasselt et al., 2020). Firstly, we performed a retrospective disproportionality analysis of PJP cases with ICIs using data from the AERSMine. Secondly, we conducted a systematic literature review to confirm whether there is an association between PJP and ICIs therapies and provide a comprehensive clinical description of PJP induced by ICIs. The flow chart of this study was displayed in Figure 1. Ethical approval was not required because this study was conducted by using deidentified data.

### 2.2 Pharmacovigilance analysis

A pharmacovigilance study was conducted from 2011 Quarter 1 (Q1, ipilimumab was approved by FDA on 25 March 2011) to 2022 Q4 with the FAERS data in AERS*Mine* to evaluate the disproportionate signal of PJP correlated with ICIs in a large-scale population. We included FDA approved 10 ICI agents (nivolumab, pembrolizumab, ipilimumab, atezolizumab, avelumab, durvalumab, dostarlimab, cemiplimab, tremelimumab, relatlimab) and one ICI combination therapies (nivolumab plus ipilimumab). PJP cases were identified by searching the Medical Dictionary for Regulatory Activities (MedDRA) (version 25.0), for preferred term “Pneumocystis jirovecii pneumonia”. Only case number more than 5 were included in this study. We collected the following information for each detected PJP reports: report year, demographic information (gender, age), drugs, indications, and co-administrated drugs. The demographics features including age and gender distribution of PJP cases associated with ICIs were investigated by comparing with all other adverse events of ICIs.

We performed the case/non-case analysis, a validated concept in pharmacovigilance, to investigate whether suspected PJP (cases) are differentially reported with immune check point inhibitors as compared to other adverse events (non-cases) (Faillie, 2019). We used two different disproportionate indicators, reporting odds ratios (RORs) and information components (IC) to reduce likelihood of false positive signals (Noguchi et al., 2021). When the lower limit of the 95% confidence interval of ROR (ROR_025_) >1 (Rothman et al., 2004) or the lower limit of the 95% confidence interval of IC (IC_025_) >0 (Bate et al., 1998), significant adverse events were detected. If the proportion of adverse events of interest is greater in patients exposed to a specific drug (cases) than in patients not exposed to this drug (non-cases), a disproportionality signal emerges, and subsequent analytical investigations are usually required before taking regulatory actions (Raschi et al., 2020). Both approaches were firstly conducted by using all other drugs in the FAERS database as a comparator, a common exploratory disproportionality analysis method.

Considering confounders such as comorbidities may affect the safety signal of PJP, we conducted primary sensitivity analyses to confirm the PJP safety signals correlated with ICIs. We excluded diseases (“renal transplant” “liver transplant” “stem cell transplant” “hiv infection” “bone marrow transplant” “inflammatory bowel disease” “organ transplant” “chronic obstructive pulmonary disease”) as PJP may occur preferentially in those immunocompromised patients with aforementioned conditions. We also excluded some drugs that cause an immunosuppression state which may further induce infection (“corticosteroids” “glucocorticoids” “immunosuppressants”). To assess the robustness of disproportionality signals and account for underlying confounders of the drug-event association, we conducted secondary sensitivity analysis by using anticancer drugs, such as traditional chemotherapy and molecular targeted therapy, as a comparator (to reduce confounding by indication and provide a clinical perspective). Firstly, we identified relevant National Comprehensive Cancer Network (NCCN) guidelines (Supplementary Figure S1), according to FDA-approved indications of ICIs. Then we extracted different cancer regimens from those selected NCCN guidelines and compared the safety signal of PJP between ICIs and traditional chemotherapy/molecular targeted therapy. Differences in categorical variables were assessed using a chi-squared test of independence performed on a 2 × 2 contingency table with Yates’ continuity correction or fisher exact test. Significance was assumed when the *p*-value less than 0.05. Data analyses were conducted by using the IBM SPSS (26.0) and Microsoft Excel (2021).

### 2.3 Systematic literature review

A comprehensive literature review was conducted through PubMed, Google Scholar, Web of Science, Embase from inception to 20 February 2023 (We have registered our protocol in PROSPERO with a number CRD42022376162 before the formal search). The search strategy included the keywords (“immunotherapy OR immune checkpoint inhibitors OR PD-1 inhibitors OR PD-L1 inhibitors OR CTLA-4 inhibitors OR nivolumab OR pembrolizumab OR cemiplimab OR dostarlimab OR atezolizumab OR avelumab OR durvalumab OR ipilimumab OR tremelimumab OR relatlimab”) AND (“Pneumocystis jirovecii pneumonia” OR “Pneumocystis pneumonia” OR “Pneumocystosis”). Mesh terms (“Immune Checkpoint Inhibitors” [Mesh]) AND “Pneumonia, Pneumocystis” [Mesh] were also used in the search process of PubMed. We only included literature written in English. Meeting abstracts were excluded. Case reports case series, case-control studies, observational studies, single-arm studies with detailed clinical information of PJP were retained in our final analyses. Two reviewers independently searched the literature and examined the relevant studies for further assessment of data, and collected clinical characteristics including age, gender, indication, absolute lymphocyte count, comorbidities, first/second-line regimens, immune-related adverse events, immunosuppressive agents, and outcome of PJP cases associated with ICIs. The quality of the reports was assessed following the recommended guidelines for publishing an adverse event report (Kelly et al., 2007).

### 2.4 Global assessment of the evidence

Although disproportionality analysis *per se* is not an estimate, it could be evaluated and used for signal prioritization, which further provides clues for regimens management clinically and regulatory actions. Previous research (Pacurariu et al., 2017) concluded criteria used for signal prioritization and of the associated decision support frameworks, including strength of evidence, public health impact, novelty and general public and media attention. This study adopted novelty of the drug event association, seriousness, and disproportionate reporting as criteria for the signal prioritization. Moreover, a causal relationship appraisal was carried out on the entire body of evidence by using adapted Bradford Hill criteria used in epidemiology that have been applied to pharmacovigilance data (van Hunsel et al., 2018; Raschi et al., 2022), including biological plausibility, strength, consistency, specificity, coherence, and analogy. The rechallenge/de-challenge item were not analyzed in this study due to missing data.

## 3 Results

### 3.1 PJP is associated with ICIs in the FAERS database

**FIGURE 2:**
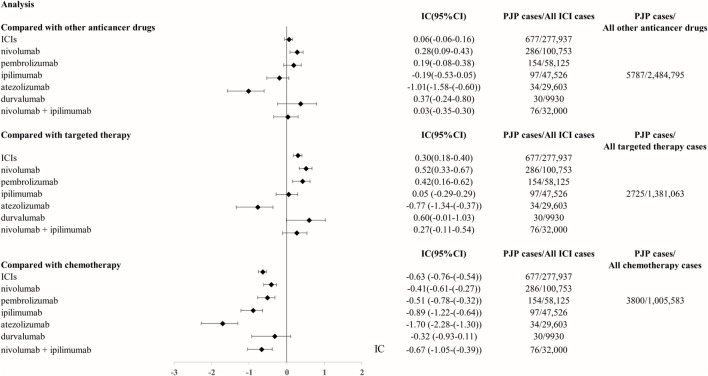
The comparison of Pneumocystis jirovecii pneumonia signal between ICIs and targeted therapy/chemotherapy in FAERS database. The list of control group (targeted therapy and chemotherapy) was extracted from NCCN guidelines for FDA approved indication of ICIs. Abbreviation: PJP, Pneumocystis jirovecii pneumonia. ROR, reporting odds ratio. IC, information component. 95%CI, 95% confidence interval. N, number. AEs, adverse events. ICIs, immune checkpoint inhibitors. NCCN, National Comprehensive Cancer Network.

From Q1, 2011 to Q4, 2022, we detected 677 patients on ICIs with PJP. In the primary analysis, we found that nivolumab, pembrolizumab, ipilimumab, atezolizumab, durvalumab, nivolumab plus ipilimumab had a significant safety signal (ROR_025_ > 1, IC_025_ > 0) for PJP (Table 1). After the sensitivity analyses, the above six ICI regimens consistently emerged with strong disproportionality, through both, ROR and IC approaches. (Table 2). When compared with all other anti-cancer drugs, nivolumab (IC_025_ 0.09, *p* = 0.001) showed significant higher safety signal of PJP. Then we found that across all ICIs (IC_025_ 0.18, *p* < 0.001), nivolumab (IC_025_ 0.33, *p* < 0.001), pembrolizumab (IC_025_ 0.16, *p* < 0.001), durvalumab (IC_025_–0.01, *p* = 0.019) presented a significant signal for PJP compared to targeted therapy. However, all ICIs showed a low signal of disproportionate reporting for PJP when compared to traditional chemotherapy (Figure 2).

**TABLE 1:** Pneumocystis jirovecii pneumonia signals of different immune checkpoint inhibitors (Primary analysis).

Drug name	N of all AEs	N of pneumocystis jirovecii pneumonia	ROR (95%CI)	IC (95%CI)
Nivolumab	100,753	286	4.91 (4.37,5.53)	2.25 (2.05,2.39)
Pembrolizumab	58,125	154	4.53 (3.86,5.32)	2.14 (1.88,2.34)
Ipilimumab	47,526	97	3.47 (2.84,4.24)	1.76 (1.43,2.01)
Atezolizumab	29,603	34	1.94 (1.39,2.72)	0.93 (0.36,1.34)
Durvalumab	9930	30	5.12 (3.57,7.33)	2.25 (1.65,2.69)
Nivolumab + Ipilimumab	32,000	76	4.03 (3.02,5.06)	1.97 (1.59,2.25)

Abbreviation: N, number; AEs, adverse events; ROR, reporting odds ratio; IC, information component; 95%CI, 95% confidence interval; The control group is all other drugs in the FAERS database.

**TABLE 2:** Pneumocystis jirovecii pneumonia signals of different immune checkpoint inhibitors after sensitivity analysis.

Drug name	N of PJP with ICIs/All AEs reports with ICIs	N of PJP with other drugs/all AEs with other drugs in the FAERS database	ROR (95%CI)	IC (95%CI)
Nivolumab	149/85,417	1885/11,066,105	10.26 (8.68,12.12)	3.22 (2.95,3.41)
Pembrolizumab	109/50,686	1925/11,100,836	12.43 (10.24,15.07)	3.49 (3.173.72)
Ipilimumab	42/38,851	1992/11,112,671	6.04 (4.45,8.20)	2.49 (1.97,2.85)
Atezolizumab	14/22,616	2024/11,128,906	2.89 (1.71,4.89)	1.44 (0.53,2.06)
Durvalumab	18/7973	2016/11,143,549	12.51 (7.36,19.90)	3.24 (2.45,3.79)
Nivolumab + Ipilimumab	36/25,638	1998/11,125,884	7.83 (5.03,10.89)	2.82 (2.26,3.21)

Abbreviation: N, number; AEs, adverse events; ROR, reporting odds ratio; IC, information component; 95%CI, 95% confidence interval; The control group is all other drugs in the FAERS database; The sensitivity analysis set could be found in the section of method.

### 3.2 Clinical features of PJP cases related to ICIs in FAERS database

**FIGURE 3:**
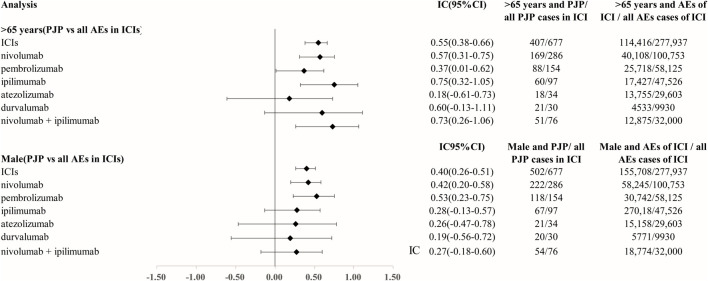
Age and gender distribution in Pneumocystis jirovecii pneumonia signal cases of ICIs. Abbreviation: PJP, Pneumocystis jirovecii pneumonia. ROR, reporting odds ratio. IC, information component. 95%CI, 95% confidence interval. N, number. AEs, adverse events. ICIs, immune checkpoint inhibitors.

We analyzed the clinical characteristics of PJP cases correlated with ICIs in the FAERS database. A total of 85.3% of PJP reports associated with ICIs were reported from 2018 to 2022.68.5% of cases were reported by health professionals. 300 of 677 (44.3%) of PJP cases died and 123 of 677 (18.2%) PJP cases experienced life-threatening situation. 66.3% (403 of 677) were elderly, age >65 years. A total of 77.0% (502/677) of PJP cases related to ICIs were males. To assess whether age >65 years is a factor may increase reporting of PJP in patients on ICIs, we calculated the ROR and IC by comparing age >65 years PJP cases/all PJP cases with age >65 years cases/all adverse events cases on ICIs. We found that elderly whose age more than 65 years old was predominant in PJP reports of overall ICIs (IC_025_ 0.38, *p* < 0.001), nivolumab (IC_025_ 0.31, *p* < 0.001), pembrolizumab (IC_025_ 0.01, *p* = 0.001), ipilimumab (IC_025_ 0.32, *p* < 0.001), durvalumab (IC_025_–0.13, *p* = 0.008), nivolumab plus ipilimumab (IC_025_ 0.26, *p* < 0.001). Similarly, we also assessed the gender distribution in PJP associated with ICIs. Males is the predominance in PJP case reports of across all ICIs (IC_025_ 0.26, *p* < 0.001), nivolumab (IC_025_ 0.20, *p* < 0.001), pembrolizumab (IC_025_ 0.23, *p* < 0.001), ipilimumab (IC_025_–0.13, *p* = 0.015), nivolumab plus ipilimumab (IC_025_–0.18, *p* = 0.029). (Figure 3). The top indications in PJP cases were lung cancer (171/667, 40.1%) and melanoma (160/677, 37.6%). We further analyzed the co-administrated regimens of ICIs in PJP cases. A total of 41.4% (280/677) cases reported to receiving glucocorticoids or corticosteroids during ICIs therapy. 69 out of 677 (10.2%) cases received immunosuppressants when they were on ICIs. Additional details of clinical features of PJP cases associated with specific ICIs regimens are shown in Table 3.

**TABLE 3:** Patient characteristics of PJP reports with immune checkpoint inhibitors in FAERS database.

Categories	Nivolumab N (%)	Pembrolizumab N (%)	Atezolizumab N (%)	Durvalumab N (%)	Ipilimumab N (%)	Nivolumab + ipilimumab N (%)	All ICIs N (%)
**Reports of PJP**	286	154	34	30	97	76	677
**Report Year**							
2016 and before	19 (6.6)	3 (1.9)	0	0	17 (17.5)	4 (5.3)	43 (6.4)
2017	30 (10.5)	13 (8.4)	0	2 (6.7)	6 (6.2)	5 (6.6)	56 (8.3)
2018	61 (21.3)	32 (20.8)	2 (5.9)	7 (23.3)	13 (13.4)	10 (13.2)	125 (18.5)
2019	52 (18.2)	27 (17.5)	4 (11.8)	9 (30.0)	9 (9.3)	9 (11.8)	110 (16.2)
2020	45 (15.7)	33 (21.4)	8 (23.5)	6 (20.0)	20 (20.6)	20 (26.3)	132 (19.5)
2021	41 (14.3)	14 (9.1)	10 (29.4)	3 (10.0)	14 (14.4)	14 (18.4)	71 (10.5)
2022	38 (13.3)	32 (20.8)	10 (29.4)	3 (10.0)	18 (18.6)	14 (18.4)	115 (17.0)
**Reporter**							
Healthcare professionals	207 (72.4)	82 (53.2)	31 (91.2)	25 (83.3)	67 (69.1)	52 (68.4)	464 (68.5)
Consumer	36 (12.6)	50 (32.5)	0	1 (3.3)	10 (10.3)	8 (10.5)	105 (15.5)
Other	43 (15.0)	22 (14.3)	3 (8.8)	4 (13.3)	20 (20.6)	16 (21.1%)	108 (16.0)
**Age Category**							
0–14	0	0	0	0	0	0	0
15–24	1 (0.4)	7 (5.3)	0	0	0	0	8 (1.3)
25–65	90 (34.6)	37 (28.0)	16 (47.1)	5 (19.2)	31 (34.1)	20 (28.2)	199 (32.4)
>65	169 (65.0)	88 (66.7)	18 (52.9)	21 (80.8)	60 (65.9)	51 (71.8)	407 (66.3)
Data available	260 (90.9)	132 (85.7)	34 (100.0)	26 (86.7)	91 (93.8)	71 (93.4)	614 (90.7)
**Gender**							
Male	222 (79.9)	118 (80.8)	21 (63.7)	20 (74.1)	67 (70.5)	54 (73.0)	502 (77.0)
Female	56 (20.1)	28 (19.2)	11 (33.3)	7 (25.9)	28 (29.5)	20 (27.0)	150 (23.0)
Data available	278 (97.2)	146 (94.8)	33 (97.1)	27 (90.0)	95 (97.9)	74 (97.4)	652 (96.3)
**Indication**							
Lung cancer	80 (40.2)	47 (73.4)	8 (72.7)	15 (100.0)	11 (14.5)	10 (16.4)	171 (40.1)
Melanoma	54 (27.1)	11 (17.2)	0	0	55 (72.4)	40 (65.6)	160 (37.6)
Renal cell carcinoma	36 (18.1)	3 (4.7)	3 (27.3)	0	10 (13.2)	11 (18.0)	63 (14.8)
Hodgkin’s disease	29 (14.6)	3 (4.7)	0	0	0	0	32 (7.5)
Data available	199 (69.6)	64 (41.6)	11 (32.4)	15 (50.0)	76 (78.4)	61 (82.4)	426 (62.9)
**Co-administration drugs**							
Glucocorticoids or Corticosteroids	123 (43.0)	41 (26.6)	19 (55.9)	12 (40.0)	49 (50.5)	36 (47.4)	280 (41.4)
Immunosuppressants	34 (11.9)	7 (4.5)	2 (5.9)	6 (20.0)	14 (14.4)	6 (7.9)	69 (10.2)
**Outcome**							
Death	141 (49.3)	69 (44.8)	13 (38.2)	12 (40.0)	36 (37.1)	29 (38.2)	300 (44.3)
Life-threatening	48 (16.8)	28 (18.2)	4 (11.8)	6 (20.0)	20 (20.6)	17 (22.4)	123 (18.2)
Disability	11 (3.8)	8 (5.2)	0	0	3 (3.1)	3 (3.9)	42 (6.2)
Hospitalization	211 (73.8)	113 (73.4)	25 (73.5)	25 (83.3)	81 (83.5)	63 (82.9)	518 (76.5)
Other Serious	262 (91.6)	122 (79.2)	13 (38.2)	16 (53.3)	86 (88.7)	70 (92.1)	569 (84.0)
**Co-reported AEs**							
interstitial lung disease	58 (20.3)	30 (19.5)	8 (23.5)	0	20 (20.6)	19 (25.0)	135 (19.9)
colitis	20 (7.0)	1 (0.6)	0	0	13 (13.4)	6 (7.9)	40 (5.9)
pneumonitis	34 (11.9)	11 (7.1)	5 (14.7)	2 (6.7)	4 (4.1)	4 (5.3)	60 (8.9)
pneumonia bacterial	3 (1.0)	10 (6.5)	4 (11.8)	0	0	0	17 (2.5)

irAEs, immune-related adverse events; ICIs, immune checkpoint inhibitors; Q3, quarter 3; Detailed list of Glucocorticoids, Corticosteroids Immunosuppressants included drugs could be found in the Supplementary File S1; Total number of outcomes for all ICIs, such as nivolumab, is not equal to total drug events (286) since patients have reported more than one outcome.

### 3.3 Published PJP case reports related to ICIs

Our literature review identified 15 PJP cases related to ICIs. 8 of 15 (53.3%) cases were lung cancer and 4 of 15 (26.7%) cases were melanoma. 8 cases (53.3%) were male while 7 cases (46.7%) were female. 8 PJP cases (53.3%) were patients with an age >60 years old. 7 of 15 (46.7%) PJP cases were co-reported with other immune-related adverse events such as colitis and hepatitis. 12 of 15 (80.0%) of cases received PD-1 inhibitors before PJP was diagnosed. 11 of 15 (73.3%) of PJP cases received glucocorticoids, corticosteroids or other immunosuppressants before PJP occurred. 5 of 15 PJP patients (33.3%) died. Additional details about ICIs regimens (and previous treatments) are listed in Table 4. The quality appraisal of the cases identified from the literature are summarized in Supplementary Figure S2.

**TABLE 4:** Summaries of published case reports of ICI-related Pneumocystis jirovecii pneumonia.

Case	Study type	Cancer	Comorbidities	Age/Gender/ALC (cells/μl)	First-line treatment	Second/Third-line treatment	irAEs	Immunosuppressive agents before PJP	Outcome
Moujaess et al. (2020)	Case report	metastatic melanoma	Hypertension. Diabetes mellitus coronary artery disease; paroxysmal atrial fibrillation	68/Male/340cells/μL	Corticosteroids and prednisone. 2000cGys irradiated	ipilimumab and Nivolumab	N/A	N/A	recovery
Arriola et al. (2015)	Case series (2 in total)	melanoma	Chronic Lymphocytic Leukemia	69/female/10,000 cells/μl	lymphadenectomy of the right axilla; Dacarbazine	ipilimumab	Colitis	steroids, infliximab, prednisolone	recovery
Arriola et al. (2015)	Case series (2 in total)	melanoma	N/A	63/female	Wide local excision and axillary block dissection	Ipilimumab	Colitis and capillary leak syndrome	steroids	recovery
Liu et al. (2020)	Case series (3 in total)	Lung squamous carcinoma	diabetes mellitus	55/Male/400 cells/μL	nivolumab	N/A	N/A	N/A	recovery
Liu et al. (2020)	Case series (3 in total)	squamous lung carcinoma	N/A	62/Male/551 cells/μL	right lower lobectomyl; radiotherapy	pembrolizumab	Radiation pneumonia (not irAEs)	intravenous methylprednisolone	deceased of respiratory failure
Liu et al. (2020)	Case series (3 in total)	advanced squamous cell lung carcinoma	N/A	62/Male/1100 cells/μL	etoposide plus cisplatin. Concurrent radiotherapy	toripalimab (PD‐1 inhibitor)	Not specified	intravenous methylprednisolone	recovery
Schwarz et al. (2019)	Case series (2 in total)	NSCLC	chronic obstructive pulmonary disease	79/Male	Carboplatin plus gemcitabine. Radiotherapy	nivolumab	Pneumonitis	corticosteroids	deceased owing to respiratory failure
Schwarz et al. (2019)	Case series (2 in total)	NSCLC	Hypercholesterolemia. Atherosclerosi	53/Male	cisplatin plus vinorelbine. Right upper lobe resection	Nivolumab plus concomitant radiotherapy	Suspected pneumonitis	high dose corticosteroid, mycophenolate mofetil + antibiotic	deceased owing to respiratory failure
Finbar Slevin et al. (2016)	Case report	Metastatic melanoma	N/A	52/Female	Vemurafenib. Right pneumonectomy	ipilimumab	terminal ileitis and pancolitis	high dose corticosteroids and infliximab	recovery
Hiba et al. (2022)	Case report	locally advanced lung adenocarcinoma	N/A	52/Female	carboplatin/paclitaxel	pembrolizumab	Suspected acute interstitial lung disease	intravenous and oral corticotherapy	deceased
Sadek et al. (2020)	Case series (2 in total)	melanoma	N/A	68/Male/0.72 cells/μL	Nivolumab. Gamma-knife radiosurgery	ipilimumab	immune-related hepatitis and colitis	methylprednisolone and infliximab	recovery
Sadek et al. (2020)	Case series (2 in total)	Hodgkin’s lymphoma	N/A	24/Female/0.21 cells/μL	Rituximab plus dexamethasone plus cytarabine plus cisplatin; bendamustine–brentuximab. Etoposide and prolonged corticosteroids	Pembrolizumab	N/A	prolonged corticosteroids; methylprednisone	Recovery
Si et al. (2021)	Case report	Primary mediastinal B-cell	N/A	18/Female/2350 cells/μL	R-DA-EPOCH radiation (mixed proton/photon), pembrolizumab, autologous stem cell transplant, BEAM	Pembrolizumab	N/A	N/A	Recovery
Feng et al. (2021)	Case report	lymphoma lung adenocarcinoma	N/A	73/Male/830 cells/μL	anti-PD1 antibody combined with pemetrexed and cisplatin	anti-PD1 antibody combined with pemetrexed	N/A	N/A	Recovery
Sanka and Hsu (2023)	Case report	NSCLC	hypertension, hyperlipidemia, depression, multiple sclerosis	53/Female	pembrolizumab, pemetrexed, and carboplatin	nab-paclitaxel	N/A	a corticosteroid dose of 50 mg prednisone equivalent (PEQ) daily	deceased

Abbreviation: ALC, ALC absolute lymphocyte; NSCLC, Non-small-cell lung carcinoma; T2DM, Diabetes mellitus type 2; R-DA-EPOCH (rituximab, etoposide, prednisone, vincristine, cyclophosphamide, hydroxydaunorubicin); BEAM (carmustine, etoposide, cytarabine, melphalan).

### 3.4 Global assessment of evidence

By the combined analysis from post-marketing adverse reports submitted to FAERS and published case reports, this study detected novel signals of PJP with ICIs and provided comprehensive evidence for the signal prioritization. Bradford Hill criteria were fulfilled, as indicated by the strength of disproportionality and its consistency throughout the analyses, thus supporting a likely causal association between PJP and ICIs (The signal priority and causal relationship appraisal could be found in Supplementary Table S2).

## 4 Discussion

To the best of our knowledge, this is the first large-scale pharmacovigilance study to investigate the association of PJP and ICIs by combining FAERS data mining and literature review. There are three main findings of this study:

Firstly, our post-marketing pharmacovigilance analysis showed that PJP was significantly associated with ICIs. Previous case reports (Arriola et al., 2015; Finbar Slevin et al., 2016; Schwarz et al., 2019; Liu et al., 2020; Moujaess et al., 2020; Sadek et al., 2020; Feng et al., 2021; Rath et al., 2021; Si et al., 2021; Hiba et al., 2022) showed that PJP may be a complication in immunotherapy. But the sample size is small. We retrospectively analyzed 677 case reports in FAERS database, conducted primary analysis and sensitivity analysis and found that all ICIs had significant higher disproportionate signals for PJP than other drugs in the FAERS database. We subsequently compared the signals of PJP between ICIs and other anticancer regimens, including targeted therapy, and chemotherapy, both were extracted from NCCN’s guideline for ICIs’ indications in order to enhance the clinical perspective of this signal comparison. Although all ICIs were detected a lower signal of PJP than chemotherapy, we found that nivolumab and pembrolizumab, both PD-1 inhibitors, showed significant higher reporting frequency for PJP than targeted therapy. And our case collections also showed that use of PD-1 inhibitors was correlated with PJP cases (12 of 15, 80.0%). However, CTLA-4 inhibitors or PD-L1 inhibitors were not associated with increased PJP reports compared with other anticancer drugs from FAERS data analysis. A previous meta-analysis (Su et al., 2019) showed that PD-L1 but not PD-1/CTLA4 inhibitors increased the risk of pneumonia compared to chemotherapy/placebo. However, another recent study (Tong et al., 2021) showed that both PD-1 and PD-L1 inhibitors significantly increase the risk of all-grade and high-grade pneumonia in NSCLC patients compared to conventional chemotherapy. Considering the conflicting research evidence, our pharmacovigilance analysis and literature collections support that PD-1 inhibitors may carry a clear potential for PJP. Further research is warranted to explore this clinical association of ICIs and PJP.

Secondly, we systematically investigated the clinical features of PJP cases associated with ICIs. Across all ICIs (which included regimens in table 1), our data analysis from FAERS database showed that males had a higher PJP report frequency than female (IC_025_ 0.26, *p* < 0.001), elderly patients >65 years had more reports than younger patients (<65 years) on ICIs (IC_025_ 0.38, *p* < 0.001). This is consistent with our literature review on published case reports (53.3% PJP cases associated with ICIs were male and age more than 65).

Thirdly, our study provides more evidence to support that PJP may be unmasked in cancer patients in the process of immune reconstitution induced by immune checkpoint inhibitors. An earlier review (Morelli et al., 2022) showed that PJP, could be categorized into a kind of opportunistic infections, and maybe associated with irAE treatment (corticosteroid, infliximab, etc.). However, there were also case reports (Inthasot et al., 2020; Feng et al., 2021) of severe pulmonary infections induced by *Mycobacterium tuberculosis*, Aspergillus fumigatus and Pneumocystis jirovecii were outside the context of immunosuppressive therapy. Our FAERS data anallysis showed that only 41.4% and 10.2% of cases received glucocorticoids/corticosteroids, or immunosuppressants during ICIs therapy, respectively. Our literature review also showed that 26.7% (4 of 15) of cases did not receive glucocorticoids/corticosteroids, infliximab, mycophenolate mofetil or other immunosuppressive agents before the occurrence of PJP. Both the FAERS data and published case reports showed that PJP cases associated with ICIs may be independent of immunosuppression. The subclinical colonization of Pneumocystis jirovecii may unmask and progress into PJP in non-HIV-infected immunosuppressed populations (Morris and Norris, 2012). Previous research (Wu et al., 2004) showed that Pneumocystis jiroveci pneumonia will be unmasked during reversal of immunosuppression in non-HIV infected patients. Emerging research (Feng et al., 2021; Lin et al., 2021) has reported that immune-related pneumonitis could be induced by immune reconstitution inflammatory syndrome. Previous preclinical study (Kauffman et al., 2021) showed that immune checkpoint blockade may cause an exaggerated immune response to fungal colonisation, which could promote fungal growth similar to recent studies in *Mycobacterium tuberculosis* infection. With the data from FAERS and published case reports, as well as previous research on mechanism of immune-related pneumonitis, we believe immune checkpoint inhibitors especially PD-1 inhibitors, may “unmask” low level PJP colonisation in the same way that antiretroviral therapy can reveal subclinical tuberculosis as immune reconstitution occurs. This is inherently counter-intuitive as developing PJP (a disease of immunocompromise) in association with ICI (which boosts immunity) but indeed be a possible mechanism for PJP.

Another study (Mansharamani et al., 2000) indicated that in immunosuppressed persons without HIV infection, CD4 + counts may be a useful clinical marker to identify specific individuals at particularly high clinical risk for PJP. Previous prospective study (Agrawal et al., 2016) showed that Absolute Lymphocyte Count <1643 μl but not 1200 μl could be the cost-effective surrogate marker for CD4 cell counts <200 cells/μL in monitoring HIV infected individuals. Moreover, a recent meeting abstract (Joshi et al., 2019) in 2019 ASCO Annual Meeting indicated that low ALC (750/μL, median) and prolonged steroid therapy are more likely to result in PJP infection as opposed to steroid therapy alone. Our case series identified 9 PJP cases with ALC amount, 7 of 9 (77.8%) had an ALC <1200 μL. The above literature provided literature evidence of monitoring ALC value as an indicator of PJP infection when CD4 T Cells amount is not accessible.

To increase the robustness of disproportionate signals detected from spontaneous reporting systems, some researchers tried to combine pharmacovigilance analysis with literature review. Wang et al. investigated fatal toxic effects associated with immune checkpoint inhibitors by using data from large academic medical centers, global WHO pharmacovigilance data, and all published ICI clinical trials (Wang et al., 2018). Stevens-Johnson syndrome/toxic epidermal necrolysis (Zhu et al., 2021) and type 1 diabetes (Zhu et al., 2022b) were found highly associated with immune checkpoint inhibitors by analyzing data from clinical trials and post-marketing data from the FAERS database. Raschi et al. (2019) confirmed that ICIs are associated with a multitude of irAEs, especially respiratory, endocrine, and hepatic toxicities by conducting parallel approach through contemporary post-marketing analysis and overview of systematic reviews. With the above research experience, this study presented that ICIs were strongly associated with PJP by the combined analysis of pharmacovigilance and systematic literature review.

Our study has several limitations. Firstly, FAERS database is a spontaneous reporting system whose data source is heterogeneous (both non-health care and healthcare practitioners) and therefore reporting bias exists. Secondly, FAERS data could not be used to calculate the incidence of PJP because of the under-reporting phenomenon and not having the full number of patients who have received the drug. Thirdly, detailed clinical information such as previous treatment regimens and stages of cancer are missing, so the reports do not confirm causality of the drug-induced event. However, this large-scale pharmacovigilance analysis did provide comprehensive information about the link between PJP and ICIs.

## 5 Conclusion

Our literature and FAERS analysis indicated that ICIs may be associated with a safety signal of PJP, especially in males aged >65years. PD-1 inhibitors emerged with a robust disproportionality signal when compared to PD-L1/CTLA-4 inhibitors as well as targeted therapy, even accounting for confounders such as concomitant immunosuppressive drugs. Anti-PD-1 therapy may unmask low level Pneumocystis jirovecii colonization and cause PJP infection in patients. More pre-clinical or clinical studies are warranted to confirm the association of PJP and ICIs and explore the potential new mechanism of ICIs-related PJP.

## Data Availability

The original contributions presented in the study are included in the article/Supplementary Material, further inquiries can be directed to the corresponding author.
