# Supplementary material for: Pneumocystis jirovecii pneumonia associated with immune checkpoint inhibitors: A systematic literature review of published case reports and disproportionality analysis based on the FAERS database
Source: Front Pharmacol. 2023 Mar 15;14:1129730. doi: 10.3389/fphar.2023.1129730 (PMC10050453; doi:10.3389/fphar.2023.1129730)
Supplement: Supplementary file 1 [file DataSheet2.docx]

**Pneumocystis jirovecii pneumonia associated with immune checkpoint inhibitors: A systematic literature review of published case reports and disproportionality analysis based on the FAERS database.**

**Supplementary file S2**

**Table S1** criteria used to identify/prioritize the signals [1].

| **Criteria** | **Details** | **Discussion** |
| --- | --- | --- |
| Strength of evidence | Strength of evidence was defined as the degree of evidence supporting a causal relationship between the drug and the event. | 1. Disproportionate reporting of PJP with ICIs in the FAERS database 2. Alternative data sources: published case reports |
| Public health impact | Public health impact was defined as the impact that a potential safety issue is likely to have on patients’ health at population level, usually through the number of patients affected by an adverse reaction and its consequences | Seriousness: high fatality rate of PJP with patients on ICIs, 44.3% in the FAERS database and 31.3% in the published case reports |
| Novelty | Novelty of drug event association referred to an association that was not previously recognized or that is not labeled in the product information of the drug. | The association of PJP with ICIs limited to case reports, this is the first large-scale pharmacovigilance study |

**Table S2.** Global assessment through adapted Bradford Hill Criteria [2].

| **Criteria** | **Description** | **Source/Method** |
| --- | --- | --- |
| Strength of the association | Although ROR and IC are not measures of risk, the strength of the disproportionality both in primary (vs. all other drugs in the FAERS database) and secondary (vs. other anticancer drugs) analyses suggests a robust signal of PJP with ICIs | Disproportionality analysis |
| Analogy | This criterion is of limited value here as there is no analogous agent to ICIs (or positive control in this study) | Literature |
| Biological plausibility/empirical evidence | Previous review [3] concluded two possible pathways of infection in ICIs therapy: immunosuppression induced opportunistic infection and dysregulated inflammatory immune response, which supports the mechanistic basis of PJP events with ICIs. | Disproportionality and literature |
| Consistency | Case reports/series have been published recently, suggesting a potential association. | Disproportionality and literature |
|  |  |  |
| Exclusion of biases/confounders^1^ | The association (statistically significant disproportionality) persisted across sensitivity analysis accounting for confounding by indication and potential co-treated immunosuppressive agents | Disproportionality |
| Specificity | Pharmacovigilance data suggest immune checkpoint inhibitors carry a stronger association for PJP as compared to other drugs. Moreover, a drug-specific effect (rather than a class-effect) cannot be excluded | Disproportionality |
| Temporal relationship | All PJP events manifested after the suspected drug was administered in both the pharmacovigilance analysis and published case reports | Post-marketing data and literature |
| Reversibility  Coherence | Data on discontinuation and reversibility are not obtained from the FAERS database but two published case reports[4, 5] showed reversibility of PJP after the de-challenge of ICIs.  The reasoning about cause and effect as present in the aforementioned criteria are in line with each other and supported by the existing knowledge. This supports the existence of a causal relationship | Literature |

1 These items were not included in the original Bradford Hill Criteria.

**Table S3.** Quality Appraisal of the Literature Reported Cases[6].

| **Lead author (citation)** | **Title** | **Patient demographics** | **Current health status** | **Medical history** | **Physical exam** | **Patient disposition** | **Drug identification** | **Dosage** | **Drug reaction interface** | **Concomitant therapy** | **Adverse events** | **Discussion** |
| --- | --- | --- | --- | --- | --- | --- | --- | --- | --- | --- | --- | --- |
| Elissar Moujaess[7] | Agree | Agree | Agree | Agree | Agree | Agree | Agree | Agree | Agree | Agree | Agree | Agree |
| Edurne Arriola[8]  (case 1) | Agree | Agree | Agree | Agree | Agree | Agree | Agree | Agree | Agree | Agree | Agree | Agree |
| Edurne Arriola[8]  (case 2) | Agree | Agree | Agree | Agree | Agree | Agree | Agree | Partially Agree | Agree | Agree | Agree | Agree |
| Ziwei Liu[9](case 1) | Agree | Agree | Agree | Agree | Agree | Agree | Agree | Agree | Agree | Partially agree | Agree | Agree |
| Ziwei Liu[9](case 2) | Agree | Agree | Agree | Agree | Agree | Agree | Agree | Agree | Agree | Agree | Agree | Agree |
| Ziwei Liu[9](case 3) | Agree | Agree | Agree | Agree | Agree | Agree | Agree | Agree | Agree | Agree | Agree | Agree |
| Michael Schwarz[10]  (case 1) | Agree | Agree | Agree | Agree | Agree | Agree | Agree | Partially agree | Agree | Agree | Agree | Agree |
| Michael Schwarz[10]  (case 2) | Agree | Agree | Agree | Agree | Agree | Agree | Agree | Partially agree | Agree | Agree | Agree | Agree |
| Finbar Slevin[11] | Agree | Agree | Agree | Agree | Agree | Agree | Agree | Partially agree | Agree | Agree | Agree | Agree |
| Zahi Hiba[12] | Agree | Agree | Agree | Agree | Agree | Agree | Agree | Partially agree | Agree | Agree | Agree | Agree |
| Maroun Sadek[13]  (case 1) | Agree | Agree | Agree | Agree | Agree | Agree | Agree | Partially agree | Agree | Agree | Agree | Agree |
| Maroun Sadek[13]  (case 2) | Agree | Agree | Agree | Agree | Agree | Agree | Agree | Partially agree | Agree | Agree | Agree | Agree |
| Si, Stephanie[5]  Yu Feng[4]  Pranavi Sanka[14] | Agree  Agree  Agree | Agree  Agree  Agree | Agree  Agree  Agree | Agree  Agree  Agree | Agree  Agree  Agree | Agree  Agree  Agree | Agree  Partially agree  Agree | Agree  Partially agree  Partially agree | Agree  Agree  Agree | Agree  Agree  Agree | Agree  Agree  Agree | Agree  Agree  Agree |

^a^The quality of reporting the cases identified from the literature was evaluated by one reviewer (SX) and cross-checked by another (MY) based on the 12 elements required by the International Society for Pharmacoepidemiology and the International Society of Pharmacovigilance for publishing adverse events reports. Possible item ratings are agree, partially, or disagree.

**References:**

1. Pacurariu AC, Coloma PM, Gross-Martirosyan L, et al. Decision making in drug safety-a literature review of criteria used to prioritize newly detected safety issues. Pharmacoepidemiol Drug Saf. 2017;26(3):327-34.

2. Raschi E, Fusaroli M, Giunchi V, et al. Adrenal Insufficiency with Anticancer Tyrosine Kinase Inhibitors Targeting Vascular Endothelial Growth Factor Receptor: Analysis of the FDA Adverse Event Reporting System. Cancers (Basel). 2022;14(19).

3. Morelli T, Fujita K, Redelman-Sidi G, et al. Infections due to dysregulated immunity: an emerging complication of cancer immunotherapy. Thorax. 2022;77(3):304-11.

4. Feng Y, Chen C, Zhao L, et al. A potential mechanism of the onset of immune-related pneumonitis triggered by anti-PD-1 treatment in a patient with advanced adenocarcinoma lung cancer: case report. BMC Pulm Med. 2021;21(1):291.

5. Si S, Erickson K, Evageliou N, et al. An Usual Presentation of Pneumocystis jirovecii Pneumonia in a Woman Treated With Immune Checkpoint Inhibitor. J Pediatr Hematol Oncol. 2021;43(2):e163-e4.

6. Kelly WN, Arellano FM, Barnes J, et al. Guidelines for submitting adverse event reports for publication. Drug Saf. 2007;30(5):367-73.

7. Elissar Moujaess EEH, Joseph Kattan. Pneumocystis Jiroveci Mimicking COVID-19 Pneumonia

in a Patient who is Receiving Ipilimumab and Nivolumab

Combination Therapy: A Case Report. Eurasian Journal of Medicine and Oncology. 2020.

8. Arriola E, Wheater M, Krishnan R, et al. Immunosuppression for ipilimumab-related toxicity can cause pneumocystis pneumonia but spare antitumor immune control. Oncoimmunology. 2015;4(10):e1040218.

9. Liu Z, Liu T, Zhang X, et al. Opportunistic infections complicating immunotherapy for non-small cell lung cancer. Thorac Cancer. 2020;11(6):1689-94.

10. Schwarz M, Kocher F, Niedersuess-Beke D, et al. Immunosuppression for Immune Checkpoint-related Toxicity Can Cause Pneumocystis Jirovecii Pneumonia (PJP) in Non-small-cell Lung Cancer (NSCLC): A Report of 2 Cases. Clin Lung Cancer. 2019;20(3):e247-e50.

11. Finbar Slevin CM, Maria Marples. Pneumocystis jirovecii pneumonia in a patient with melanoma treated with infliximab and corticosteroids for ipilimumab-associated colitis. Global Dermatology. 2016.

12. Hiba Z, Abdelmoughit H, Zaynab IH, et al. Pneumocystis pneumonia in patient with lung adenocarcinoma: early side effects from pembrolizumab. Radiol Case Rep. 2022;17(10):3979-81.

13. Sadek M, Loizidou A, Drowart A, et al. Pneumocystis Infection in Two Patients Treated with Both Immune Checkpoint Inhibitor and Corticoids. J Immunother Precis Oncol. 2020;3(1):27-30.

14. Sanka P, Hsu A. A Case of Pneumocystis jirovecci in a Patient with Non-Small Cell Lung Cancer Treated with Immunotherapy. R I Med J (2013). 2023;106(1):11-3.
