# Supplementary material for: Pneumocystis jirovecii pneumonia associated with immune checkpoint inhibitors: A systematic literature review of published case reports and disproportionality analysis based on the FAERS database
Source: Front Pharmacol. 2023 Mar 15;14:1129730. doi: 10.3389/fphar.2023.1129730 (PMC10050453; doi:10.3389/fphar.2023.1129730)
Supplement: Supplementary file 2 [file DataSheet1.docx]

**Title:**

**Pneumocystis jirovecii pneumonia associated with immune checkpoint inhibitors: A systematic literature review of published case reports and disproportionality analysis based on the FAERS database.**

**Supplementary File 1**

**Part one NCCN guidelines related to ICIs’ indications in this study.**

1.Bladder Cancer, Version 3.2020, NCCN Clinical Practice Guidelines in Oncology ;PMID: 32135513

2. Breast Cancer, Version 3.2020, NCCN Clinical Practice Guidelines in Oncology

PMID: 32259783

3. Colon Cancer, Version 2.2021, NCCN Clinical Practice Guidelines in Oncology

PMID: 33724754

4. Esophageal and Esophagogastric Junction Cancers, Version 2.2019, NCCN Clinical Practice Guidelines in Oncology ; PMID: 31319389

5. Gastric Cancer, Version 2.2022, NCCN Clinical Practice Guidelines in Oncology

PMID: 35130500

6. Head and Neck Cancers, Version 2.2020, NCCN Clinical Practice Guidelines in Oncology ;PMID: 32634781

7. Hepatobiliary Cancers, Version 2.2021, NCCN Clinical Practice Guidelines in Oncology; PMID: 34030131

8. Hodgkin Lymphoma, Version 2.2020, NCCN Clinical Practice Guidelines in Oncology; PMID: 32502987

9. Melanoma, Version 2.2016, NCCN Clinical Practice Guidelines in Oncology

PMID: 27059193

10. Merkel Cell Carcinoma, Version 1.2018, NCCN Clinical Practice Guidelines in Oncology; PMID: 29891526

11.NCCN Guidelines® Insights: B-Cell Lymphomas, Version 5.2021; PMID: 34781267

12. NCCN Guidelines Insights: Cervical Cancer, Version 1.2020; PMID: 32502976

13. NCCN Guidelines Insights: Kidney Cancer, Version 1.2021; PMID: 32886895

14. NCCN Guidelines Insights: Malignant Pleural Mesothelioma, Version 3.2016

PMID: 27407123

15. NCCN Guidelines® Insights: Melanoma: Cutaneous, Version 2.2021; PMID: 33845460

16. NCCN Guidelines Insights: Small Cell Lung Cancer, Version 2.2018; PMID: 30323087

17. NCCN Guidelines Insights: Uveal Melanoma, Version 1.2019 ;PMID: 32023525

18. NCCN Guidelines Updates: Management of Metastatic Colorectal Cancer

PMID: 31117039

19.Uterine Neoplasms, Version 1.2018, NCCN Clinical Practice Guidelines in Oncology; PMID: 29439178

20. NCCN Guidelines Insights: Non-Small Cell Lung Cancer, Version 2.2021

PMID: 33668021

**Part two drug list of causing an immunosuppression state.**

1. Glucocorticoids or corticosteroids:
   1. **corticosteroids:** "beclometasone" "prednisone" "dexamethasone" "flunisolide" "budesonide" "betamethasone" "tixocortol" "fluticasone" "mometasone" "triamcinolone" "fluticasone furoate" "ciclesonide" "dexamethasone, combinations" "fluticasone, combinations"
   2. **glucocorticoids:** "betamethasone" "dexamethasone" "fluocortolone" "methylprednisolone" "paramethadione" "prednisone" "prednisolone" "glucocorticoids" "triamcinolone" "hydrocortisone" "cortisone" "prednylidene" "rimexolone" "deflazacort" "cloprednol" "meprednisone" "cortivazol"
2. **Immunosuppressants**
   1. **"selective immunosuppressants":** "muromonab-cd3" "antilymphocyte immunoglobulin (horse)" "antithymocyte immunoglobulin (rabbit)" "mycophenolic acid" "sirolimus" "leflunomide" "alefacept" "everolimus" "gusperimus" "efalizumab" "natalizumab" "abatacept" "eculizumab" "belimumab" "fingolimod" "belatacept" "tofacitinib" "teriflunomide" "apremilast" "vedolizumab" "alemtuzumab" "begelomab" "ocrelizumab" "baricitinib" "ozanimod" "emapalumab" "cladribine" "imlifidase" "siponimod" "ravulizumab" "upadacitinib" "filgotinib" "itacitinib" "inebilizumab" "belumosudil" "peficitinib" "ponesimod" "anifrolumab" "ofatumumab" "teprotumumab" "pegcetacoplan" "sutimlimab" "deucravacitinib" "ublituximab" "efgartigimod alfa" "avacopan"
   2. **tumor necrosis factor alpha (tnf-alpha) inhibitors**: "etanercept" "infliximab" "adalimumab" "certolizumab pegol" "golimumab"
   3. **interleukin inhibitors**: "daclizumab" "basiliximab" "anakinra" "rilonacept" "ustekinumab" "tocilizumab" "canakinumab" "secukinumab" "siltuximab" "brodalumab" "ixekizumab" "sarilumab" "sirukumab" "guselkumab" "tildrakizumab" "risankizumab" "satralizumab" "netakimab" "bimekizumab" "spesolimab" "olokizumab"
   4. **calcineurin inhibitors**: "ciclosporin" "tacrolimus" "voclosporin"
   5. **other immunosuppressants**: "azathioprine" "thalidomide" "methotrexate" "lenalidomide" "pirfenidone" "pomalidomide" "dimethyl fumarate" "darvadstrocel" "diroximel fumarate"
